# Choroidal Metastasis as a Presenting Feature of Lung Cancer in a Patient Presenting With Sudden-Onset Bilateral Loss of Vision: A Case Report

**DOI:** 10.7759/cureus.101518

**Published:** 2026-01-14

**Authors:** Jolomi Y Alele, Subramanian Dinakaran, Sarah Chatharoo, Adelola Adekolu-John, Pankaj Chaturvedi

**Affiliations:** 1 Internal Medicine, Sheffield Teaching Hospitals, Sheffield, GBR; 2 Opthalmology, Doncaster and Bassetlaw Teaching Hospitals NHS Foundation Trust, Doncaster, GBR; 3 Diabetes and Endocrinology, Doncaster and Bassetlaw Teaching Hospitals NHS Foundation Trust, Doncaster, GBR

**Keywords:** cancer, chest x-ray, choroidal metastases, copd, ct chest, fundoscopy, lung, sudden vision loss

## Abstract

Choroidal metastases are a rare but significant presentation of lung cancer, often indicating advanced disease and associated with poor prognosis. This report describes a 61-year-old woman with chronic obstructive pulmonary disease and a heavy smoking history who presented with sudden bilateral vision loss. Ophthalmic evaluation revealed bilateral choroidal metastases with exudative retinal detachment. Imaging identified a right hilar lung mass with widespread metastases. Due to her poor clinical status, biopsy was not feasible, and she received palliative care. She died within two weeks of presentation. This case highlights the importance of considering ocular symptoms as possible initial signs of occult and usually advanced systemic malignancy, necessitating a thorough history taking, examination and relevant investigations to identify distant malignancy and what treatment options may be offered to the patient.

## Introduction

Choroidal metastases are a rare but clinically significant presentation of lung cancer. Although the choroid represents the most frequent site of intraocular metastases [1], the incidence of intraocular metastases estimated from post-mortem examination is 12%, with the most common metastatic foci being the choroid [2]. Breast cancer is the most common primary malignancy, followed by lung cancer [2]. Clinically, patients may present with blurred vision, photopsia, visual field defects, or even profound vision loss, which can severely impact quality of life. In some cases, choroidal metastases may be bilateral, and their presence often signifies advanced disease and a poor prognosis.

The diagnostic challenge lies in the fact that ocular symptoms may precede the detection of systemic malignancy. In such scenarios, ophthalmologists play a crucial role in raising suspicion for an underlying primary tumour and initiating appropriate systemic evaluation. This is particularly relevant in individuals with risk factors such as a significant smoking history, where lung cancer remains a leading primary cause.

We present this case because it highlights the importance of considering choroidal metastases as a potential initial manifestation of lung cancer. The case underscores the need for heightened clinical suspicion, as timely recognition can lead to earlier systemic workup and diagnosis. Furthermore, the uniqueness of this report lies in its bilateral involvement and brevity of the clinical course in this patient which adds important clinical insight for physicians.

## Case presentation

We present a 61-year-old woman with chronic obstructive pulmonary disease with a 50-pack-year smoking history who presented to the accident and emergency department with a sudden onset of painless bilateral loss of vision. She reported a history of active smoking but denied any additional respiratory symptoms other than a longstanding smoker’s cough, which had not changed in character prior to admission. She had a past medical history of a tight stenosis of the left subclavian artery just distal to its origin, for which she was being conservatively managed under the vascular surgeons. 

**Figure 1:**
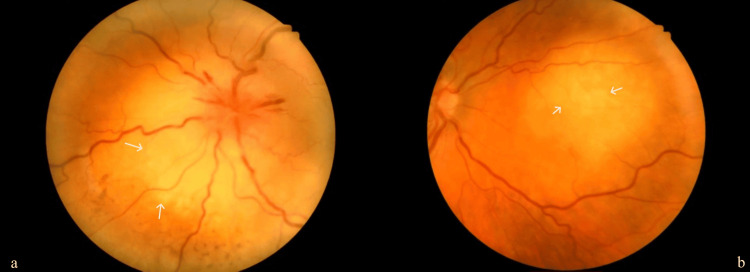
Fundoscopic image of left eye (a) and right eye (b), with pale elevations representing metastatic lesions

**Figure 2:**
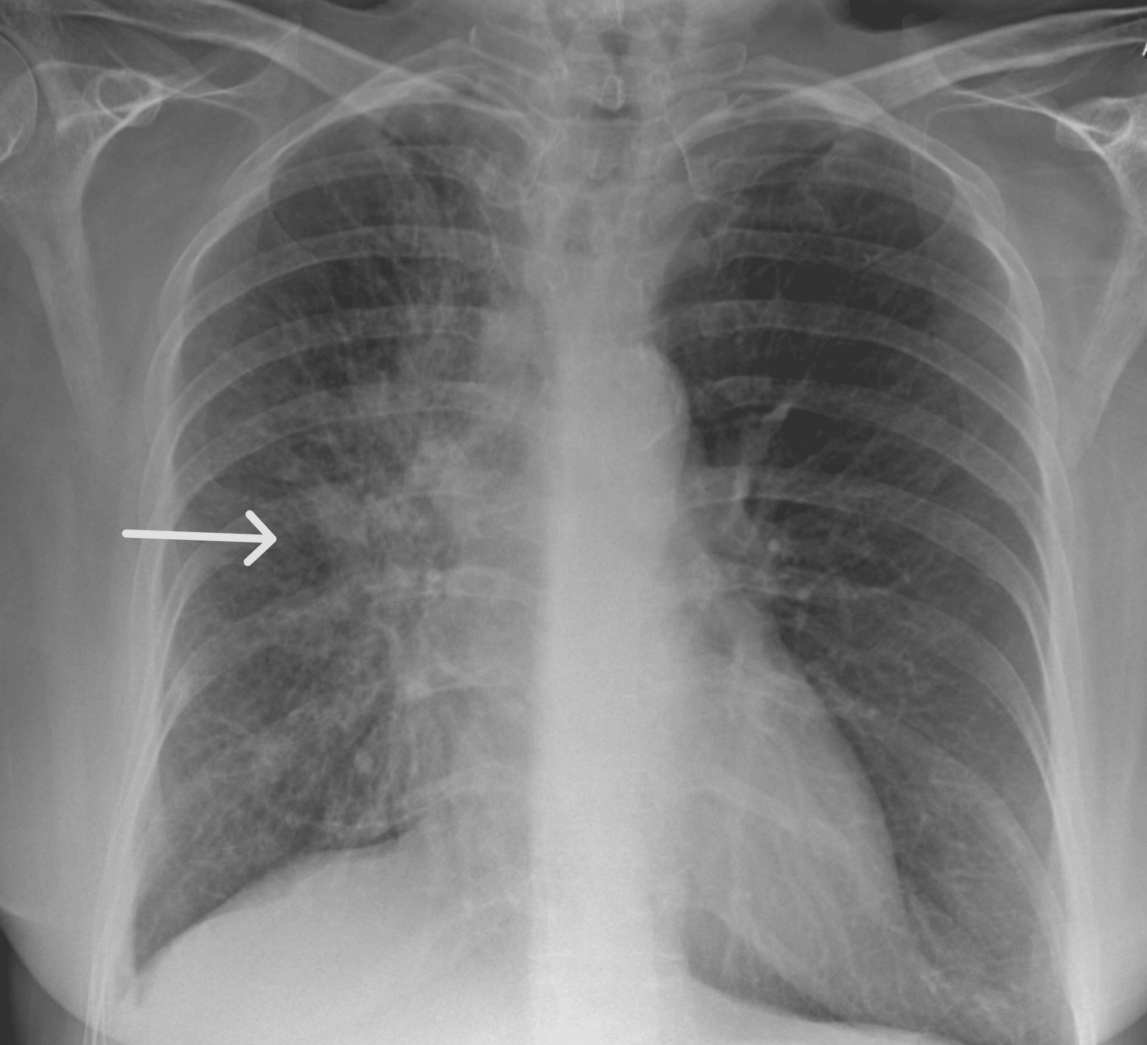
Initial chest X-ray showing a bulky right hilum

**Figure 3:**
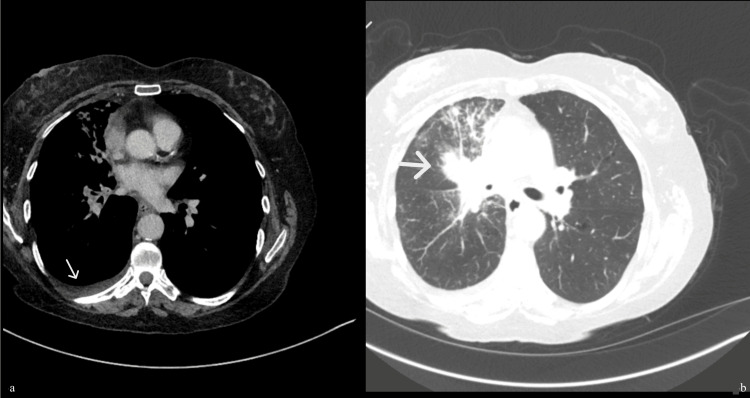
Computed tomography (CT) thorax abdomen and pelvis lung view in the axial section showing right-sided unilateral pleural effusion (a) and right hilar mass (b).

The ophthalmology team reviewed her, and an ophthalmic examination showed vision with no perception of light in both eyes. Fundus examination revealed suspicious multiple choroidal metastatic lesions in both eyes, with a large exudative retinal detachment in the left eye (Figures 1a, 1b). Given her smoking history, she was evaluated further with a chest X-ray, which showed a very bulky right hilum (Figure 2). Due to the suspicion of a lung malignancy as a likely primary in this case, she was further evaluated with a computed tomography scan (CT scan) of her chest, abdomen and pelvis which showed a 30mm right hilar mass with lymphangitis extending into the right upper and middle lobes, early changes in the right lower lobe and a small right-sided pleural effusion (Figures 3a, 3b).  The tumour encased the right main bronchus and extended into the mediastinum with mediastinal lymphadenopathy. There was omental and peritoneal tumour infiltration with multiple small retroperitoneal nodes. Her computed tomography (CT) head showed no abnormality.  Figures 1-3 show images of the fundoscopy findings, initial chest X-ray, and CT chest, abdomen and pelvis.

She later developed a blue, pulseless left upper limb; vascular surgeon's opinion was a suspicion of tumour spread, worsening her already poor baseline blood supply from stenosis of the left subclavian artery at the origin. It was then decided she was for the best supportive care; she had supportive care from the palliative care team aimed at pain and breathlessness, psychological and emotional support. She unfortunately died within two weeks of hospital admission.

Due to deterioration in the patient’s clinical state, she could not undertake any invasive investigation or procedure such as a bronchoscopy or pleural aspiration. For the same reason, a planned magnetic resonance imaging (MRI) of the head was not performed. A post-mortem examination was not undertaken as we could not obtain consent.

## Discussion

The choroid is the most common intraocular site for metastatic disease; it has been postulated that this is due to its rich vascular supply [1]. The most common primary site of metastases to the choroid is the breast, followed by the lungs [2]. For lung cancer, the most common histological subtype is adenocarcinoma, followed by squamous cell cancer, small cell cancer, and large cell cancer [3,4].

Metastases to the choroid can present as unifocal and unilateral or bilateral and multifocal, with the former being more associated with metastatic lung cancer and the latter being more associated with metastatic breast cancer [1]. In our case, however, the patient presented with bilateral findings from disseminated lung cancer.

Presenting features of choroidal metastases depend on the location and degree of invasion of the lesion [3] and can range from blurred vision, reduced visual acuity and visual field loss being the most common reported presenting features to less common features such as floater, pain, red eye, raised intraocular pressure, metamorphosia and diplopia, with some patients having no symptoms [3,5]. In our case, the patient presented with a sudden onset of bilateral loss of vision.

The differential diagnosis of choroidal metastases includes choroidal melanoma, choroidal granuloma, sclerochoroidal calcification, choroidal osteoma, choroidal hemangioma, choroidal neovascularisation, and posterior scleritis [1,5]. As some cases, such as our case, present without a history of a known primary cause, diagnosis can pose a challenge; however, specific features of choroidal metastases on ophthalmoscopy and other imaging modalities, as will be discussed below, can help differentiate choroidal metastases from other choroidal tumours [1].

On fundoscopy, choroidal metastases appear as a flat or raised, uni or multifocal mass, typically in the posterior pole with various characteristics that depend on the primary malignancy's nature [3,4]. The lesion is more frequently yellow or white, although it could be orange in primary from renal cell cancer, carcinoid tumour or thyroid cancer, or brown-grey in metastases from melanoma [1]. Furthermore, brown or dark pigment representing lipofuscin can often be seen scattered on the lesion's surface, giving it a leopard skin appearance [3,5].

Several investigation modalities are available to help characterise the lesions and differentiate them from close differentials. Autofluorescence is useful in characterising the tumour surface and monitoring tumour margin progression. Choroidal metastases on autofluorescence show hypoautofluorescence of the tumour with overlying areas of bright 3+ hyperautofluorescence, which depict deposits of lipofuscin, and 2+ hypoautofluorescence, which depict subretinal fluid [1]. Ultrasound helps differentiate choroidal metastases from close differentials such as choroidal melanomas. On ultrasound, choroidal metastases show high reflectivity on A-scans and appear echodense on B-scans in contrast to melanomas, which show low reflectivity on A-scans and are acoustically hollow on B-scans [1]. On fluorescein angiography, choroidal metastases display an early arterial phase hypofluorescence with progressive hyperfluorescence in the late venous phase [1]. Furthermore, on fluorescein angiography, choroidal metastases show multifocal retinal pigment epithelial pinpoint leaks [6]. On MRI, the lesions appear as well-demarcated masses, which are iso-intense in T1-weighted images and hypointense in T2-weighted images [1]. Optical coherence topography is another investigation modality that shows anterior compression or obliteration of the overlying choriocapillaries, an irregular (lumpy bumpy) anterior contour, and posterior shadowing [5]. In cases of unknown primary or diagnostic uncertainty, FNAB for cytological analysis is useful [6].

In addition to the above, a systematic assessment is necessary in patients with choroidal metastasis, especially where there wasn't a known primary as in our case, these would include tumour markers, mammography, CT, MRI and PET scan of head, thorax abdomen and pelvis to search for a primary source of malignancy and extent of dissemination.

Treatment of choroidal metastases depends on the patient's functional status, number of lesions, location and laterality [1]. As most cases of choroidal metastases are found in advanced disease, treatment options should be carefully discussed with patients and their families, with the treatment goal being focused on maintaining quality of life [7]. Systemic treatment is the preferred treatment option for patients with bilateral multifocal choroidal metastases; focal therapy is advised in tumours presenting with visual loss or unresponsive to systemic treatment [1]. Options for systemic therapy include chemotherapy, immunotherapy, or hormone therapy, and the choice of treatment is largely dependent on the type of primary cancer; for example, the use of tamoxifen or aromatase inhibitors in choroidal metastases from breast cancer, as they tend to express oestrogen or progesterone receptors [1]. In a study by Yang et al., 75% of patients treated with chemotherapy for choroidal metastases from non-squamous cell lung cancer showed tumour regression [8]. EBRT (external beam radiotherapy) is a treatment modality at a 40- 60 Gy dose, with reported tumour regression in 85-93% of patients [1]. Anti-VEGF injections are another treatment option with successful management of metastases from breast, lung, and colorectal cancer reported. Other treatment options include gamma knife radiosurgery, proton beam radiotherapy, and photodynamic therapy [1]. Interestingly, in a treatment of 36 patients reported by Shields et al., with plaque radiotherapy, there was noted immediate regression in 100% of patients; this lasted in as much as 94% of patients over a mean follow-up period of 11 months [6]. Unfortunately, like in our case, at the time of presentation and diagnosis, some patients may be too unwell with poor functional status to have any form of therapy.

Choroidal metastases are associated with poor prognosis as they tend to become obvious late in the course of malignancy and are often associated with advanced disease and dissemination [9]. The median survival time from diagnosis to death has been cited at six months, with a range of 0.5 to 47 months [5]. In our case, the patient was unfortunately at the lower end of the range.

## Conclusions

In conclusion, this case illustrates a rare presentation of lung cancer manifesting as bilateral choroidal metastases with acute vision loss. The prognosis in such cases remains poor and therapeutic options are limited. It underscores the need for clinicians to consider ocular symptoms as potential markers of underlying malignancy and highlights the importance of a thorough systemic evaluation, particularly in patients without a prior cancer diagnosis, as patients who are fit enough may benefit from targeted or systemic therapies aimed at improving quality of life.
